# Topotecan Microneedle Scleral Patch: A Transscleral Drug Delivery Study for Retinoblastoma

**DOI:** 10.1016/j.xops.2026.101226

**Published:** 2026-05-11

**Authors:** Vishal Raval, R. Arawindh, Sharayu Naik, Saumya Jakati, Venkata Vamsi Krishna Venuganti

**Affiliations:** 1 Department of Pharmacy, Birla Institute of Technology and Science (BITS) Pilani, Hyderabad, Telangana, India; 2 The Operation Eyesight Universal Institute for Eye Cancer, L V Prasad Eye Institute, Hyderabad, Telangana, India; 3 Ophthalmic Pathology Laboratory, L V Prasad Eye Institute, Hyderabad, Telangana, India

**Keywords:** Retinoblastoma, Topotecan, Microneedle scleral patch, Bioavailability

## Abstract

**Purpose:**

We aimed to design, fabricate, and evaluate the pharmacokinetic properties of topotecan loaded with microneedle scleral patch (MSP) in rabbit eye.

**Methods:**

The MSP was fabricated using a 3-dimensional printed master mold. The second step involved making polydimethylsiloxane mold using a Sylgard 184 Silicone Elastomer Kit. The MSP was fabricated using a high-molecular-weight sodium hyaluronate biopolymer containing 90 conical microneedles (MNs) in a 15 × 6 array format. The MSP was loaded with 100 μg of topotecan to study its pharmacokinetics across choroid–retina complex.

**Results:**

The dimensions of MNs were uniform across the array measuring 548 ± 3.7 μm length, 336 ± 7.5 μm width, and 18 μm tip diameter. In the *ex vivo* goat eye model, the required insertion force was 0.026 N per needle, which was 50 times lower than the compression strength of 1.27 N per needle. The MNs were inserted up to a depth of 225 μm. In the *in vivo* rabbit model (9 eyes), topotecan levels peaked at 1 hour (2.75 ± 2 μg/g) and decreased at 2 hours (0.64 ± 0.3 μg/g), attaining 200 times the therapeutic target level. At 8 hours, the drug level was undetectable (0.02 ± 0.01 μg/g). The patch completely dissolved within 2-4 minutes with unchanged fundus appearance and no retinal toxicity.

**Conclusions:**

A single topotecan-loaded MSP (100 μg) achieved highly selective retinal tissue distribution, with a retinal-to-plasma ratio of 275-fold, which was 4.7-fold higher than intra-arterial chemotherapy (58.9) and 209-fold higher than intravenous chemotherapy (1.32), supporting its potential benefit for RB treatment.

**Financial Disclosure(s):**

Proprietary or commercial disclosure may be found in the Footnotes and Disclosures at the end of this article.

Retinoblastoma (RB) is the most common intraocular tumor in children, accounting for approximately 11% of cancers occurring in the first year of life, with 95% of cases diagnosed before the age of 5 years.^1^ In the United States, the reported incidence of RB in children aged 0 to 4 years is 12.4 cases per million,^1^^,^^2^ and India contributing to 1500-2000 cases per year.^3^ In developed countries, advances in treatment strategies have significantly improved the success of vcsual salvage alongside globe preservation. In contrast, in underdeveloped countries, intraocular tumors often remain undiagnosed until advanced stages, posing a serious threat to globe salvage; these regions account for >90% of children affected by RB.^4^^,^^5^ The key to globe salvation and vision preservation in RB is early diagnosis and appropriate treatment.^6^

In the last 2 decades, intravenous chemotherapy (IVC), which reduces tumor bulk before focal therapies, has been the mainstay of RB treatment.^7^^,^^8^ However, IVC is known to cause systemic toxicity including neutropenia, infections, ototoxicity, and secondary cancers later in life.^9^ To achieve higher globe salvage rates, since 2008, there has been a trend toward adopting intra-arterial chemotherapy (IAC), in which supra-selective delivery of chemotherapeutics is achieved by a microcatheter advanced to the ophthalmic artery via access from the femoral artery. The direct delivery of chemotherapeutic agents results in higher concentrations in tumors and minimizes systemic absorption, allowing the use of melphalan, a highly effective chemotherapeutic agent against RB that cannot be used systemically because of its myelosuppressive toxicity.^10^^,^^11^ Indeed, reported outcomes for advanced intraocular RB (group D–E eyes) are more favorable with IAC than with IVC.^12^ The globe salvage rates have ranged from 36% to 100% for group D eyes^13^, ^14^, ^15^, ^16^ and 17% to 87% for group E eyes.^13^^,^^14^^,^^17^ In a systematic review of published studies, the overall globe salvage rate was 74% with IAC as a first-line treatment modality.^18^

Compared with IVC, IAC is safe with fewer systemic side effects; however, local transient side effects, such as redness, eyelid edema, loss of eyelashes, and ptosis, usually resolve within 2 to 3 months,^19^ while permanent vision-threatening intraocular vascular complications, such as choroidal occlusive vasculopathy, vitreous hemorrhage, and ophthalmic artery occlusion, remain a concern.^20^ Furthermore, intravitreal chemotherapy (IVitC) is associated with complications such as transient ocular discomfort, redness, vitreous hemorrhage, retinal detachment, temporary rise in intraocular pressure, and risk of infection.^21^

There is an unmet need to identify new routes for delivering chemotherapeutic agents near the target site. We hypothesize that the transscleral route vis a vis a suprachoroidal route is the most suitable route of administration of chemotherapeutic agents to the posterior segment of the eye. The application of drugs within the sclera will allow their diffusion to reach the target site through the sclera–choroid–retinal barrier. The suprachoroidal space is the region between the choroid and sclera, bound anteriorly by the scleral spur and posteriorly by the optic nerve.^22^ Subsequently, a minimally invasive suprachoroidal injection was developed as an alternative method of delivery into the suprachoroidal space using an off-the-shelf syringe and an engineered short needle.^23^^,^^24^ Patel et al^25^ demonstrated the *ex vivo* feasibility of injecting particle-containing suspensions into the eyes of animals and humans. In another study, administration of sodium fluorescein via suprachoroidal, posterior subconjunctival, and intravitreal injection to Sprague–Dawley rats resulted in the highest amounts in the choroid–retina over 6 hours.^26^ The maximum concentration was much greater in the choroid–retina than in the vitreous or anterior chamber after suprachoroidal injection. Based in part on these findings, clinical trials have explored the efficacy and safety of suprachoroidal administration of triamcinolone acetonide in conditions affecting the posterior segment.^27^, ^28^, ^29^ In pursuit of delivering drugs close to the target area (retina/choroid), suprachoroidal delivery of chemotherapy would be an ideal route to access choroidal circulation,^24^^,^^30^ bypassing the ophthalmic artery and avoiding undesirable effects of IAC.^22^

Topotecan hydrochloride is classified under class I of the Biopharmaceutical Classification System, indicating good aqueous solubility and membrane permeability.^31^ Topotecan is a potent inhibitor of DNA topoisomerase-I, with a half maximal inhibitory concentration (IC_50_) value of 14 ng per milliliter (ng/mL) in cell cultures and animal studies of RB.^32^ Intravitreal administration of topotecan results in trace plasma concentrations, thereby minimizing systemic exposure and avoiding identifiable systemic toxicity.^33^ The safety and toxicity profile of topotecan is well established in both animal and clinical studies delivered by IVitC injections of 100 μg in 0.1 mL injections given 2 to 4 weeks or with multiple cycles of IAC.^34^, ^35^, ^36^, ^37^, ^38^, ^39^, ^40^ High-dose intravitreal topotecan showed no retinal toxicity, anterior inflammation, or systemic effects in rabbit RB models.^38^ Histopathology confirmed preserved retinal architecture and electroretinogram stability, with no plasma accumulation observed.^39^ It is a naturally fluorescent chemical, allowing fluorometric and photographic display of the distribution^41^^,^^42^ in addition to measurements using high-performance liquid chromatography (HPLC) technique.^34^^,^^41^

Microneedle-based ocular patches have been under various stages of clinical development for vaccine and drug delivery and have recently been studied for their effectiveness in drug delivery to the eye.^43^^,^^44^ Preliminary studies by our group have demonstrated that rapidly dissolving microneedle (MN)-based ocular patches can be developed to effectively deliver water-soluble antibiotics, water insoluble antifungal agents, and corticosteroids using both MN corneal patches and microneedle scleral patches (MSPs).^45^, ^46^, ^47^ When analyzed using stereomicroscope and scanning electron microscope, these MN patches showed MN dissolution with drug delivery.^45^^,^^48^^,^^49^ Microneedle scleral patch would rapidly dissolve within the scleral tissue, allowing removal of the base plate (devoid of needles) within 2 minutes. The polymer matrix allows the release of topotecan, thereby achieving high durability and therapeutic concentrations.

Considering the high cost of IAC and the lack of acceptance of the alternative (enucleation), we propose the development of a dissolvable polymer-based MSP that is cost-effective and easy to administer. This study aimed to design, fabricate, and characterize topotecan-loaded MSP and further evaluate its ocular pharmacokinetics and biodistribution for safety, efficacy, and toxicity in an *in vivo* rabbit model. We will test the working hypothesis that the application of dissolvable polymer-based MSP will achieve a therapeutic concentration above IC_50_ of >14 ng/mL in retina/choroid tissue for 4 hours (similar to that achieved with IAC).

## Methods

### Materials

Aqua Grey 8K resin was procured from Phrozen Technology. Isopropyl alcohol was purchased from Finar. Sylgard 184 Silicone Elastomer Kit was obtained from Dow Corning. Topotecan hydrochloride was procured from TCI Chemicals Ltd. Sodium hyaluronate (molecular weight 1200 kDa) was purchased from Catalyst. The HPLC column (Kinetex core-shell, 5 μm C18, 100 Å, 250 × 4.6 mm) was procured from Phenomenex. The HPLC-grade acetonitrile, methanol, and glacial acetic acid were obtained from Finar. Ammonium acetate extra pure was purchased from SRL.

### Master Mold Design and 3-Dimensional Printing

The master mold layout was generated using AutoCAD 2024. An array of 15 × 6 cone-shaped MNs was designed with dimensions of 715 μm length, 305 μm base width, and 20 μm tip width, with an interneedle spacing of 300 μm. The baseplate dimensions were 12.77 × 7.33 × 2 mm (length × width × height), enclosed in a rectangular shell (22.77 × 17.33 × 6 mm, length x width x height; thickness 0.8 mm; 5 mm spacing from baseplate). The master mold design was saved in .dwg and exported in. stl format. Postdesign, the .stl file was imported into CHITUBOX software for preprocessing. Support structures were generated using the software to ensure structural stability during the bottom-up printing process. The master mold design was sliced at a resolution of 10 μm layer thickness and exported in .ctb format for fabrication. The design was printed using a Phrozen Sonic Mini 8K 3-dimensional (3D) printer. After 3D printing, the supports were removed from the master mold and washed with isopropyl alcohol and kept for post-ultraviolet curing using the Phrozen cure kit.

### Preparation of Polydimethylsiloxane Mold

The 3D-printed master mold was used to cast the polydimethylsiloxane mold. Sylgard 184 elastomer and curing agent were mixed (9:1 w/w), vortexed for 10 minutes, and degassed in a vacuum drier. The mixture was poured into the master mold and cured at room temperature for 2 hours, followed by 3 hours at 70°C. The cured mold was collected, washed with methanol and water, vacuum-dried, and examined under a stereomicroscope for defects.

### Fabrication of Topotecan-Loaded MSP

**Figure 1:**
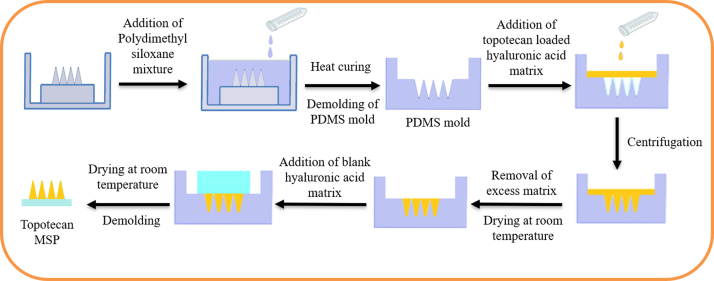
Schematic representation of casting of the topotecan-loaded MSP. MSP = microneedle scleral patch; PDMS = polydimethylsiloxane.

Figure 1 shows a schematic representation of topotecan-loaded MN patch preparation. A polymer matrix comprising 3% w/w Topotecan HCl and 7% w/w sodium hyaluronate in deionized water was prepared and degassed by centrifugation (10 000 rpm, 10 minutes). The matrix was applied to polydimethylsiloxane mold cavities and centrifuged (4500 rpm, 5 minutes). Excess polymer was removed, and the mold was dried at room temperature. This process was repeated 5 times to ensure complete cavity filling. Residual traces of the drug polymer were removed using a wet tissue.

For the backing layer, an 11% w/w sodium hyaluronate blank polymer matrix was applied as a 200 μm layer and centrifuged (4500 rpm, 2 minutes). Two additional blank polymer layers (500 μm each, 2 hours apart) were added. The mold was dried for 24 hours at room temperature and demolded to obtain the drug-loaded MSP.

### Characterization of Topotecan-MSP

Microneedle dimensions were assessed using a stereomicroscope (SZX7, Olympus) at 1×, 2.5×, and 5× magnifications. Scanning electron microscope imaging was performed after sputter-coating with gold (10 nm; EM ACE200, Leica Microsystems) at 1000 to 10 000× magnification.

Mechanical strength was measured using a texture analyzer (Stable Microsystems). Microneedle arrays were compressed using a 10-mm Delrin probe at 0.1 mm/s up to 0.5 mm displacement. Force–displacement curves were recorded. Insertion tests were performed on the excised goat sclera using similar conditions. Pre- and posttest MN morphology was imaged microscopically.

Fourier-transform Infrared Spectroscopy (Ultima IV, Rigaku) was used to study drug–polymer interactions. Powder X-ray diffraction analysis (Rigaku IV) was performed for neat topotecan, polymer, physical mixtures, and MSP (scan range 5°–40°, step 2°/minutes, resolution 1.5 Å). Differential scanning colorimetry (DSC 60, Shimadzu) was used for thermal analysis (25°C–500°C, 10°C/minute). Thermogravimetry analysis (DTG-60, Shimadzu) was performed for moisture content determination (25°C–500°C, 10°C/minute).

Rhodamine-loaded MSP penetration into goat sclera was visualized using confocal laser scanning microscopy (excitation: 545 nm, emission: 566 nm; step size 5 μm up to 300 μm; 10× objective).

### HPLC Method Development for Topotecan HCl

Quantification of topotecan HCl was performed using HPLC (Phenomenex) with a Kinetex C18 column. The mobile phase consisted of 0.02 M ammonium acetate buffer (pH 4) and acetonitrile (82:18 v/v), flow rate 0.7 mL/min, and injection volume 5 μL. Column temperature was maintained at 25°C. A fluorescence detector was set at excitation and emission wavelengths of 380 and 536 nm, respectively. Calibration standards (1–1024 ng/mL) were prepared from a stock solution (1 mg/mL in methanol).

### Drug Content Analysis in MSP

Microneedles and backing layers were dissolved separately in 1 mL of deionized water and 4 mL methanol-HCl (1000:1 v/v). Samples were homogenized (12 000 rpm, 5 minuts) and centrifuged (10 000 rpm, 10 minutes). Supernatant was analyzed by the HPLC method described earlier.

### *In Vitro* Dissolution of Topotecan-MSP

Microneedle scleral patch was dipped in phosphate buffer (pH 7.4) in a 24-well plate, ensuring only the needles contacted the medium. Microneedle scleral patch was removed and examined by scanning electron microscope to assess MN dissolution at different time intervals of 5, 10, 15, 20, and 25 seconds.

### Bioanalytical Method Development

Topotecan extraction from the ocular tissues was achieved using the protein precipitation method. The dissected tissue samples were homogenized in cold methanol-HCl (1000:1 v/v, 1:5 w/v). Topotecan-spiked homogenates (10–1280 ng/mL) were prepared from primary standards. After centrifugation (12 000 rpm, 15 minutes), the supernatant was analyzed by the HPLC method under the same chromatographic conditions. Calibration curves were generated to assess linearity and correlation.

### *Ex Vivo* Scleral Permeation

Franz diffusion cells (PermeGear) were assembled and filled with phosphate buffered saline (pH 7.4, 37°C, stirred) in the receptor compartment. The goat sclera was mounted between the donor and receptor compartment, equilibrated for 30 minutes, and observed for water loss. Microneedle scleral patch was applied to the episcleral site with thumb pressure for 1 minute, and mounted without air bubbles. For comparison, 100 μg of topotecan HCl solution in 200 μL was added to the donor compartment. Samples (300 μL) from the receptor compartment were withdrawn at predetermined intervals (0–24 hours) and replaced with fresh buffer. All samples were analyzed by the HPLC method described earlier.

### *Ex Vivo* Ocular Biodistribution

The excised goat eye globes were divided into 2 groups, intravitreal injection (0.1 mg/0.1 mL) and MSP application (100 μg) group. Microneedle scleral patch was applied with a 3D-printed applicator for 15 minutes. Eyes were dissected at 1 and 4 hours, and the tissue samples (sclera, cornea, retina–choroid, vitreous, aqueous humor, and lens) were homogenized in cold methanol-HCl (1:5 w/v), centrifuged (12 000 rpm, 15 minutes), and analyzed by the HPLC method described earlier.

### *In Vivo* Ocular Biodistribution

Male New Zealand white rabbits (2–2.5 kg) were acclimatized for 7 days prior to the study. Animals were anesthetized with ketamine (35 mg/kg) and xylazine (5 mg/kg) administered intramuscularly. Formulation groups included MSP (100 μg topotecan HCl applied to the sclera, 5 mm posterior to limbus for 10 min) and intravitreal injection (50 μg in 50 μL). The residual topotecan in the patch was quantified after 10 minutes application.

Blood samples were collected from the marginal ear vein from 0.25 to 8 hours. At 1, 2, and 8 hours after MSP application, rabbits were euthanized using an overdose of thiopentone sodium (125 mg/kg intravenously), followed by cervical dislocation. Eyes were enucleated, dissected, and the tissues (cornea, aqueous humor, lens, vitreous, choroid–retina, and sclera) were processed as described earlier to extract topotecan and analyzed by the HPLC method. Retinal fundus photographs were obtained using the iCare DRS plus TrueColor confocal fundus camera at 40% true color exposure setting before and after application to assess the safety of the formulations.

### Evaluation of the Safety of MSP Using OCT

The safety of the scleral tissue was evaluated using OCT (IXanner AEAR). OCT images of the superior anterior sclera were obtained at multiple time points during MSP application, specifically at 0, 15, 30, and 60 minutes, to assess the depth of penetration of MNs on the sclera.

### Histological Assessment of Ocular Tissues after MSP Application

Immediate histological assessment of scleral integrity was performed by applying MSP on the rabbit right eye sclera (study eye) for 2 minutes, after which the animal was euthanized, following which both study and control eye (left eye) were enucleated within 5 minutes. Both the enucleated globes were dissected fresh; right eye was dissected along with the attached MSP. The sectioned callottes embedded in optimal cutting temperature media and rapidly frozen. Cryosections were cut at 5 μm thickness, fixed in 70% isopropyl alcohol, and rapidly stained with hematoxylin and eosin. Sections were further dehydrated, cleared, and mounted using distyrene, plasticizer, and xylene mountant. Imaging was performed using a digital image scanner (Leica, Aperio AT2).

Delayed safety assessment of MSP was performed using enucleated globes obtained 1 hour after MSP application. The eyes were fixed for 24 hours in modified Davidson fixative at room temperature, then processed, trimmed at 3 levels (nasal, central, and temporal), and paraffin-embedded. Sections (∼5 μm) were stained with hematoxylin and eosin and cover-slipped for evaluation of histopathological changes in the retina and choroid at the application site and the opposite quadrant.

### Statistical Analysis

All the results were presented as mean ± standard deviation. The sample size ranged from 3 to 6. The experimental groups were compared with analysis of variance or Student *t*-test (GraphPad prism V8.0), where a *P* value of ≤0.05 was considered statistically significant. All animal experiments were performed under the auspices of the BITS Pilani, Hyderabad campus, Hyderabad after obtaining a prior approval from an Institutional Animal Ethics Committee, which operates under the guidelines of the Committee for the Purpose of Control and Supervision of Experiments on Animals.

## Results

### Characterization of Topotecan-MSP

**Figure 2:**
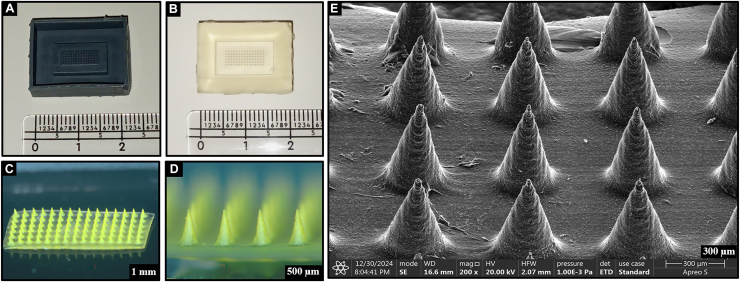
Representative digital images of the master mold **(A)**, PDMS mold **(B)**, and stereomicroscopic images of the topotecan MSP at different magnifications **(C and D)**, scanning electron microscopic images of the topotecan MSP **(E)**. MSP = microneedle scleral patch; PDMS = polydimethylsiloxane.

Microneedle dimensions were measured using a stereomicroscope with a calibrated scale. Figures 2A–D show digital images of the master mold, polydimethylsiloxane mold, and stereomicrographs of the topotecan MSP. The MNs exhibited an average height of 548 ± 4 μm, base width of 336 ± 8 μm, and tip width of 18 ± 2 μm. Scanning electron microscope images of the topotecan MSP were presented in Figure 2E.

**Figure 3:**
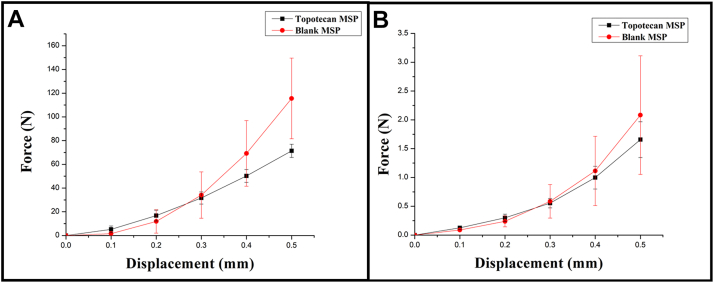
Force-displacement curves obtained after compression test of blank and topotecan MSP **(A)**, and insertion test of blank and topotecan MSP into the excised goat sclera **(B)**. MSP = microneedle scleral patch.

Mechanical testing demonstrated compression strengths of 116 ± 28 N for blank MSP and 71 ± 5.6 N for topotecan MSP (Fig 3A). Force–displacement analysis during insertion on the excised sclera showed insertion forces of 2.36 ± 1.03 N (blank MSP) and 1.66 ± 0.31 N (topotecan MSP) (Fig 3B).

### Physicochemical and Solid-State Characterization of Topotecan-MSP

**Figure 4:**
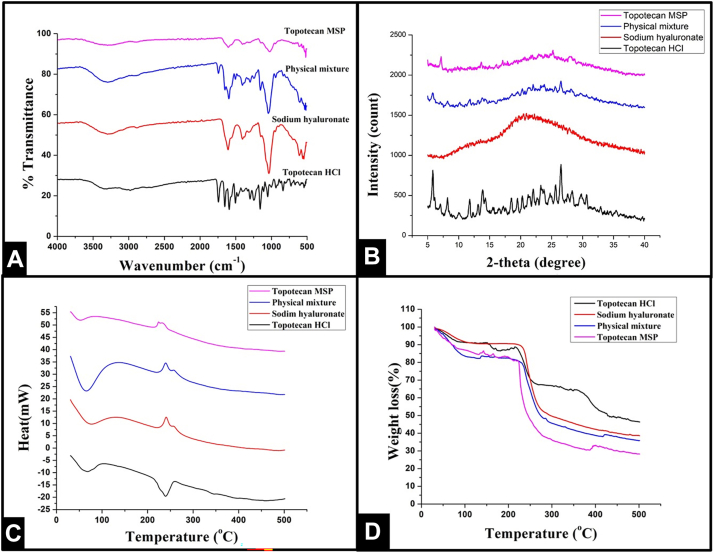
Representative Fourier-transform infrared spectra **(A)**, powder X-ray diffraction peak pattern **(B)**, differential scanning colorimetry thermograms **(C)**, and thermogravimetry analysis thermograms **(D)** of pure topotecan HCl, sodium hyaluronate, physical mixture (topotecan HCl + sodium hyaluronate), and topotecan MSP. The physical mixture was prepared with topotecan HCl: sodium hyaluronate ratio same as that in topotecan MSP. MSP = microneedle scleral patch.

Fourier-transform infrared spectra confirmed the presence of characteristic peaks for topotecan HCl at 3344 cm^–1^ (aromatic O–H stretching), 2993 cm^–1^ (alkyl C–H stretching), 1743 cm^–1^ (lactone C=O stretching), 1654 cm^–1^ (amide C=O stretching), and 1595 cm^–1^ (aromatic C=C stretching). Sodium hyaluronate exhibited peaks at 3297 cm^–1^ (O–H stretching), 2886 cm^–1^ (alkyl C–H stretching), and 1608 cm^–1^ (amide C=O stretching). Distinctive peaks of both compounds were observed in the physical mixture and topotecan-loaded MSP (Fig 4A).

Powder X-ray diffraction patterns confirmed the crystalline nature of topotecan HCl, with prominent peaks at 2θ values of 7.11°, 8.36°, 14.80°, 24.67°, 27.68°, and 30.97°. Sodium hyaluronate showed no diffraction peaks, indicating an amorphous structure. The physical mixture exhibited semicrystalline features, while topotecan-MSP showed only a few weak peaks at 7.45°, 10.07°, 16.03°, 22.92°, and 30.74° (Fig 4B).

Differential scanning calorimetry thermograms revealed an endothermic transition for topotecan HCl at 228.9°C, confirming its crystalline nature. Sodium hyaluronate exhibited a glass transition between 40 and 100°C and crystallization at ∼250°C. No distinct endothermic peak of topotecan HCl was observed in the physical mixture or topotecan MSP, suggesting drug–polymer interactions (Fig 4C).

Thermogravimetric analysis demonstrated no significant weight loss for topotecan HCl up to 240°C, after which stepwise mass loss was observed. Sodium hyaluronate showed weight loss of 9% (30°C–100°C), 50% (223°C–297°C), and 8% (297°C–500°C). The physical mixture lost ∼80% of its weight in a triphasic pattern, while topotecan MSP exhibited 92% weight loss across 30°C to 500°C (Fig 4D).

### Microneedle Insertion and Channel Formation

**Figure 5:**
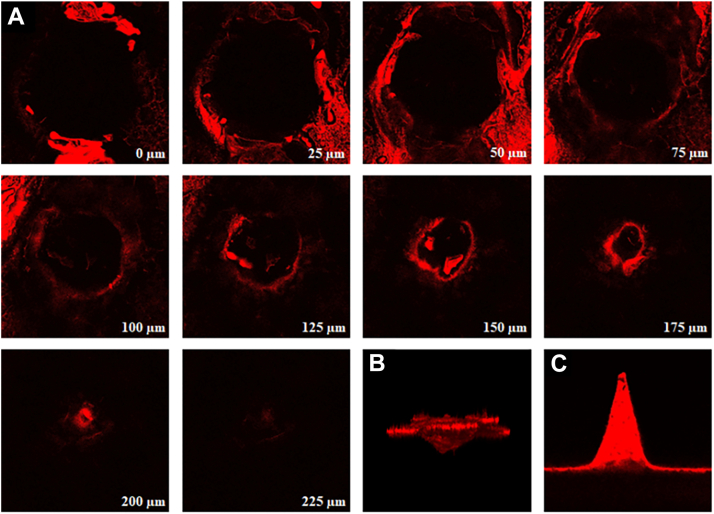
Confocal micrographs of the sclera's optical sections captured from surface (0 μm) to its interior (225 μm) **(A)**, 3D representation of the microchannel formed because of the insertion of rhodamine-loaded MSP **(B)**, and image of rhodamine-loaded MSP before scleral insertion **(C)**. 3D = 3-dimensional; MSP = microneedle scleral patch.

A 3D model of microchannels formed after insertion of rhodamine-loaded MSP, along with confocal images of scleral optical sections (0–225 μm depth), confirmed an insertion depth of 225 μm into the excised goat sclera (Fig 5).

### Drug Content in MSP

Figure S1 (available at www.ophthalmologyscience.org) shows the overlay of chromatograms and calibration curve for topotecan HCl. The method was linear over a concentration range of 2 to 1024 ng/mL (*R*^*2*^ = 0.9998). The retention time of topotecan was 6.5 min. High-performance liquid chromatography analysis showed that each MSP contained 121 ± 8.1 μg of topotecan HCl. Of this, 103 ± 9.6 μg was localized within 90 MNs, while 17.6 ± 4.0 μg was retained in the baseplate.

### *In Vitro* Dissolution

**Figure 6:**
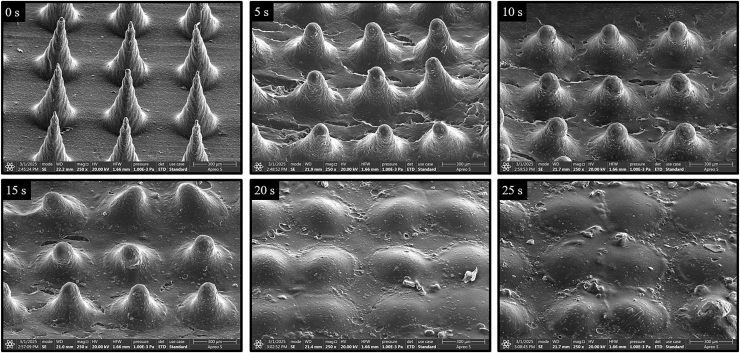
Scanning electron microscopic images of *in vitro* dissolution of topotecan MSP at different time points of 0 second, 5 seconds, 10 seconds, 15 seconds, 20 seconds, and 25 seconds. MSP = microneedle scleral patch.

Figure 6 shows scanning electron microscope images of progressive dissolution of MNs in phosphate buffer (pH 7.4) at 0, 5, 10, 15, 20, and 25 seconds. Microneedle height decreased with increasing time, with ∼95% dissolution observed at 25 seconds.

### *Ex Vivo* Scleral Permeation

**Figure 7:**
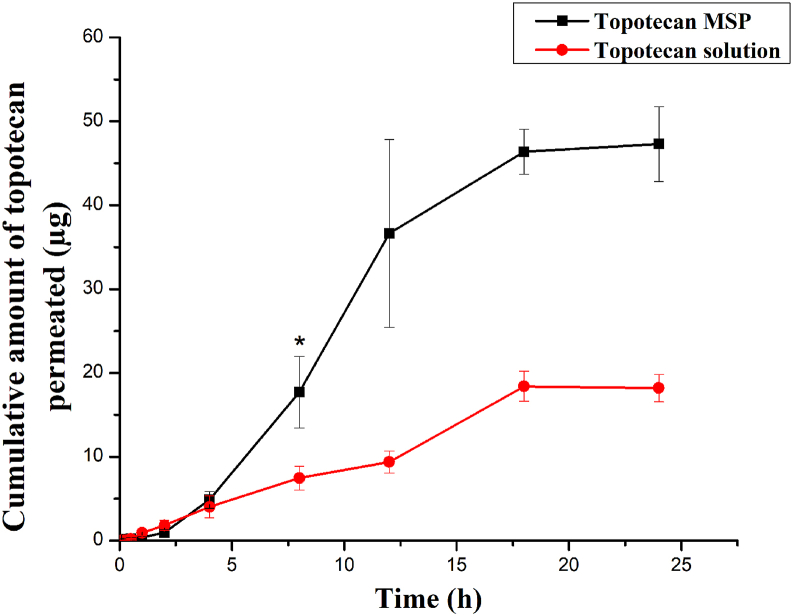
Cumulative amount of topotecan permeated across the excised goat sclera after administration of the MSP and topotecan solution. Asterisk (∗) represents that the value is significantly different at *P* < 0.05 compared with topotecan solution. MSP = microneedle scleral patch.

Table 1 shows the permeation parameters for topotecan permeation across the excised goat sclera. Compared to topotecan solution (1.54 ± 0.81 μg/cm^2^/hour), the flux of topotecan delivered by MSP was significantly higher (4.8 ± 0.55 μg/cm^2^/hour). Both permeability and diffusion coefficients were greater for MSP than for solution group (Table 1). Figure 7 illustrates cumulative permeation profiles across the excised goat sclera.

**Table 1:** Permeation Parameters for Topotecan Transport through the Excised Goat Sclera

Parameters	Topotecan Solution	Topotecan-MSP
Cumulative amount permeated (μg/cm^2^)	28.6 ± 2.6	74.4 ± 7.0
Flux (μg/cm^2^/hr)	1.5 ± 0.8	4.8 ± 0.6
Lag time (hr)	0.8 ± 0.5	1.8 ± 0.3
Permeability coefficient (cm/hr)	0.015 ± 0.001	0.048 ± 0.005
Diffusion coefficient (cm^2^/hr)	0.0006 ± 0.0003	0.0014 ± 0.0002

MSP = microneedle scleral patch.

### *Ex Vivo* Biodistribution

**Figure 8:**
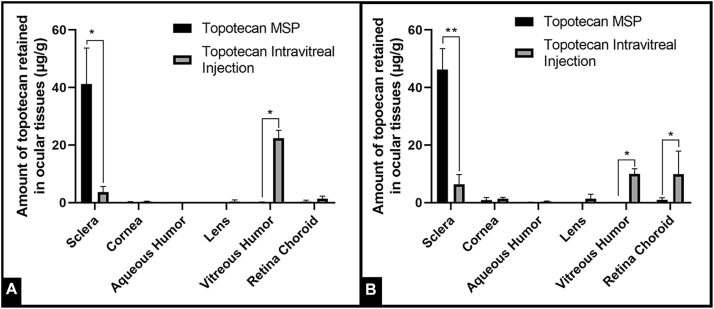
*Ex vivo* distribution of topotecan in various ocular tissues after application for 1 hour **(A)** and 4 hours **(B)**. MSP = microneedle scleral patch.

Figure S2 (available at www.ophthalmologyscience.org) shows the experimental setup for biodistribution studies in the excised goat eyes using MSP and intravitreal injection. Figure 8 shows topotecan levels in ocular tissues at 1 hour and 4 hours. Topotecan concentration in the sclera after MSP application was 11-fold higher than after intravitreal injection. In the retina–choroid complex, topotecan concentration was 0.41 μg/g at 1 hour and increased further at 4 hours after MSP application.

### *In Vivo* Biodistribution

Using rabbit ocular tissues, a bioanalytical method for topotecan HCl was validated. Calibration curves were linear over 10 to 1280 ng/mL (*R*^*2*^ > 0.99), with a lower limit of quantitation of 5 ng/mL, ensuring adequate sensitivity for ocular bioanalysis.

*In vivo* biodistribution was evaluated in New Zealand white rabbits using MSP and intravitreal injection. High-performance liquid chromatography calibration ensured quantification down to 5 ng in the ocular tissues. Microneedles were successfully inserted using a 3D-printed applicator, and the baseplate was removed after 10 minutes. The patch fully dissolved within 2 to 4 minutes without altering retinal fundus appearance.

**Figure 9:**
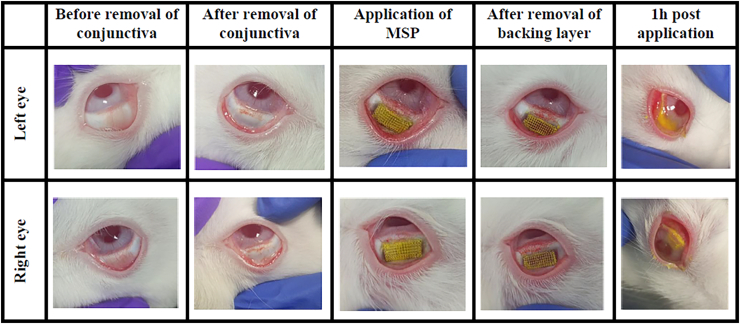
Representative digital images of rabbit eyes taken before, during, and after the MSP application. MSP = microneedle scleral patch.

**Figure 10:**
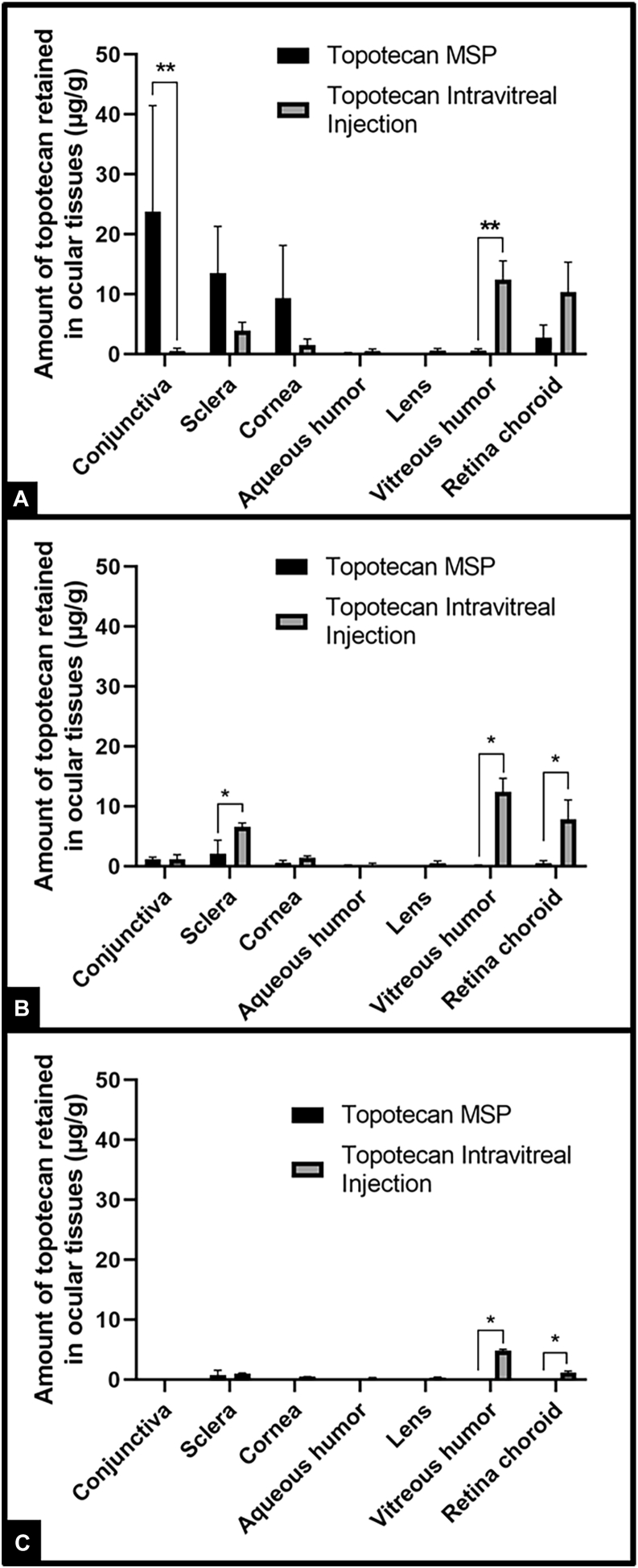
Topotecan distribution in different ocular tissues in rabbit eyes at different time points, including 1 hour **(A)**, 2 hours **(B)**, and 8 hours **(C)**, after intravitreal injection and MSP application. MSP = microneedle scleral patch.

**Figure 11:**
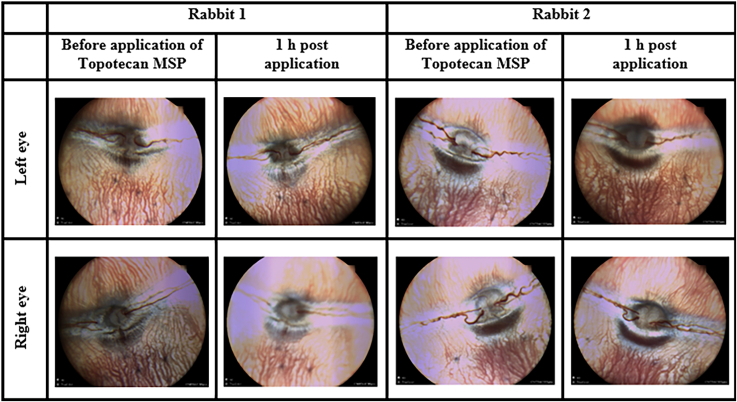
Retinal fundus images of rabbit ocular model before and after application of topotecan MSP. MSP = microneedle scleral patch.

After the MSP application, transient conjunctival redness was observed at 1 hour but resolved within the next hour (Fig 9). Tissue distribution analysis showed that topotecan levels in the choroid–retina complex peaked at 1 hour (2.75 ± 2 μg/g), decreased at 2 hours (0.64 ± 0.3 μg/g), and were nearly undetectable by 8 hours (0.02 ± 0.01 μg/g) (Fig 10). Plasma concentrations were low: 0.016 ± 0.009 μg/mL (1 hour), 0.017 ± 0.001 μg/mL (2 hours), and 0.030 ± 0.006 μg/mL (8 hours). The concentration of the topotecan in the vitreous humor was 0.55 ± 0.34 μg/g (1 hour), 0.14 ± 0.1 μg/g (2 hours), and 0.07 ± 0.02 μg/g (8 hours). The concentration of the topotecan in the aqueous humor was 0.1 ± 0.09 μg/g (1 hour), 0.16 ± 0.12 μg/g (2 hours), and 0.011 ± 0.01 μg/g (8 hours). Application of MSP preserved posterior segment integrity and did not disrupt retinal structures, with no apparent clinical signs of retinal toxicity (Fig 11).

### Evaluation of the Safety of MSP Using OCT

**Figure 12:**
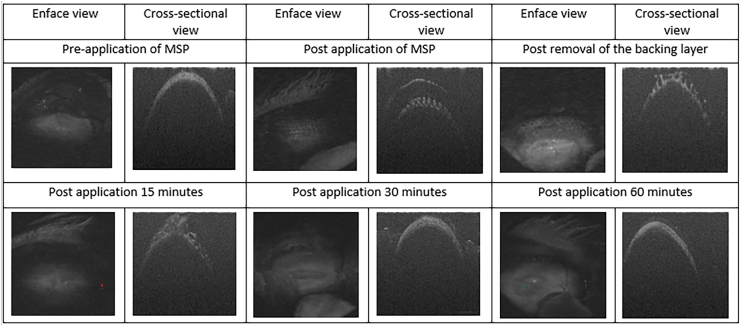
Serial OCT images of the superior region of the anterior sclera of the rabbit during application of MSP at 0, 15, 30, and 60 minutes. MSP = microneedle scleral patch.

OCT was performed at 0, 15, 30, and 60 minutes after application of the MSP, demonstrating only transient and reversible tissue changes. Baseline imaging showed normal scleral contour and reflectivity. At 15 minutes, a localized depression was noted at the MN insertion site, consistent with mechanical indentation rather than true tissue damage. By 30 minutes, partial restoration of the scleral contour was observed, and by 60 minutes, the scleral surface had returned to near-baseline appearance, indicating complete structural recovery. Figure 12 shows the serial OCT images of the superior region of the anterior sclera of the rabbit during application of MSP at 0, 15, 30, and 60 minutes.

### Histological Assessment of Ocular Tissues after MSP Application

The histopathological assessment was conducted twice; first assessment was immediately after the application of the MSP and second after 1 hour postapplication.

**Figure 13:**
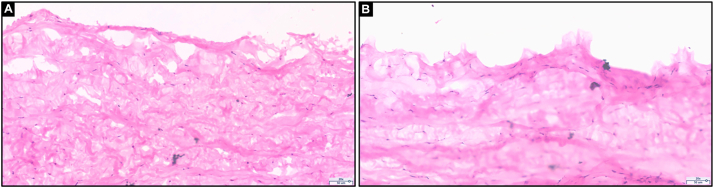
Histological evaluation of excised scleral tissue. **A,** Control showing normal lamellar collagen architecture. **B,** Post-MSP application demonstrating preserved scleral structure without fiber disruption, necrosis, or inflammatory cell infiltration, consistent with superficial mechanical indentation without true tissue injury. MSP = microneedle scleral patch.

In eyes subjected to immediate enucleation after MSP application (within 5 minutes), the study eye demonstrated discrete, well-delineated focal serrations and depressions at the MN insertion site compared with the control eye. These surface serrations corresponded to a peak-to-peak distance of approximately 300 μm, mirroring the interneedle spacing on the curved superficial scleral interface. Histopathologic evaluation revealed an intact scleral structure, with preservation of the orderly lamellar architecture of collagen fibers. There was no evidence of fiber disruption, stromal clefts, necrosis, or inflammatory cell infiltration. The observed changes were limited to superficial scleral contour alterations, consistent with transient mechanical indentation rather than changes induced by true tissue injury (Fig 13).

**Figure 14:**
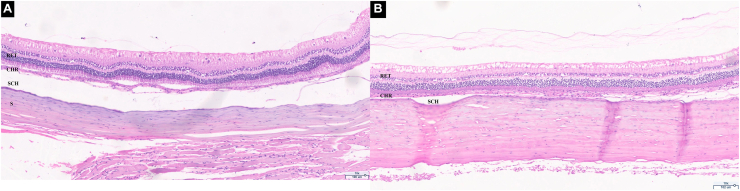
Histological sections showing preserved retinal, choroidal, suprachoroidal, and scleral architecture at the MSP application site **(A)** and opposite quadrant **(B)**. The retina demonstrates intact lamination without edema or structural disruption; the choroid shows preserved vascular architecture without congestion or hemorrhage; the suprachoroidal space is unremarkable. The sclera maintains normal lamellar collagen organization with no evidence of inflammation or cellular infiltration. MSP = microneedle scleral patch.

On delayed histological assessment (1 hour postapplication), no significant microscopic alterations were identified in the retina or choroid, either at the MSP application site or in the opposite quadrant, indicating preservation of tissue integrity across both regions. Detailed evaluation of the retinal layers demonstrated intact lamination without evidence of edema, cellular disorganization, necrosis, or disruption of the inner or outer retinal architecture. The choroid similarly exhibited preserved vascular architecture, with no signs of vascular congestion, thrombosis, hemorrhage, or stromal disruption. The suprachoroidal space remained unremarkable, without expansion or fluid accumulation. Importantly, the sclera showed maintained lamellar architecture of collagen organization with no evidence of fiber disruption or structural compromise. There were no histological features suggestive of inflammation, edema, or vascular dilatation within the scleral or adjacent tissues (Fig 14).

## Discussion

In individuals with RB, several methods, including systemic IVC, IAC, periocular, and IVitC injections, can be used to deliver drugs to the posterior segment of the eye. Recently, a novel method for administering chemotherapeutic drugs to the posterior segment of the eye via the suprachoroidal route has been investigated.^50^^,^^51^ Suprachoroidal injection has several potential benefits over IVitC and IAC methods, including more targeted posterior segment drug delivery, bypassing vascular access risks of IAC while avoiding direct vitreous contamination from IVitC.^24^ Several other novel drug-delivery strategies targeting the posterior segment have been evaluated in preclinical and clinical studies, including subconjunctival topotecan embedded in fibrin sealant, nanotechnology-based drug-delivery systems, and episcleral chemoplaques to deliver chemotherapeutics in RB.^52^, ^53^, ^54^

Considering the features of these drug delivery techniques, MNs have been suggested as a promising minimally invasive substitute.^43^^,^^55^, ^56^, ^57^ This method has been investigated as a potential treatment for conditions affecting the anterior and posterior segments of the eye. Coated, dissolvable, and hollow MNs hold great promise for the delivery of drugs into the eyes.^25^^,^^44^^,^^47^^,^^55^^,^^56^^,^^58^ Microneedles are typically applied in ocular treatment to the cornea, sclera, or suprachoroidal space, allowing drugs to bypass corneal or scleral barriers and be deposited within the tissues or released into the anterior or posterior segment.^30^^,^^55^^,^^56^^,^^59^, ^60^, ^61^, ^62^, ^63^, ^64^, ^65^, ^66^ We have developed a topotecan MSP that can be applied to the sclera in a minimally invasive manner. Microneedle scleral patch application allows the release of chemotherapeutic agents within the sclera and diffuses through the choroid and reaches the retina where the RB originates. The novelty of the present work lies in 2 main areas: ease of chemotherapeutic administration, and localized drug delivery, resulting in fewer adverse effects.

Topotecan-loaded MSP may offer several potential advantages over IVC and IAC, as this approach is expected to minimize systemic toxicity because of low plasma drug levels while simultaneously achieving higher drug concentrations within the choroid and retina—levels that may not be attainable with intravitreal injection alone. The tissue levels of topotecan can be best depicted as a ratio to the plasma level, accounting for variations in drug dose, concentration, volume, route of administration, and animal model, which emphasizes selective tissue distribution. The vitreous and aqueous humor drug levels are not considered critical for preclinical evaluation of MSP-loaded topotecan, as these compartments could be supplemented through established intravitreal or intracameral delivery if necessary. Together, these findings support further preclinical investigation of MSP as a localized strategy for retinal tumor treatment.

The clinical efficacy of topotecan for intraocular RB has been well established via various delivery routes, including IAC, IVitC, and IVC, with the exception of peribulbar injection, and targets both ocular compartments (vitreous cavity and anterior chamber). Retinal drug concentration is critical because RB originates in the retina and progresses internally toward the vitreous and externally toward the choroid. The mean retinal concentration achieved after the application of topotecan-loaded MSP was (2.75 [0.67–4.83]) μg/g of tissue, which was comparable to that of IAC (4.28 [3.55–11.4]) μg/g of tissue and substantially higher than that of IVC (0.064 [0.030–0.084] μg/g of tissue). Peak retinal levels (2.75 [0.67–4.83]) μg/g of tissue) declined rapidly by 60 minutes (0.64 [0.24–1.04] μg/g of tissue), followed by a slower decline until 480 minutes (0.026 μg/g of tissue). Retinal topotecan concentrations remained approximately 34 to 345-fold higher than the IC_50_ (14 ng/g of tissue) and 5.3 to 38-fold higher than the 90% inhibitory concentration (IC_90_) (126 ng/mL) for RB cell inhibition throughout the 480 min study duration, indicating pharmacologically effective exposure. The results of our study were comparable to those of a recently published proof-of-concept study of suprachoroidal injection of topotecan (50 μg/0.05 mL) for the treatment of RB establishing the selective tissue distribution of topotecan lactone moiety (retina/plasma, 1377.8), which was 23-fold higher than that reported with IAC (58.9) and more than 1000-fold higher than IVC (1.32). The retinal levels were nontoxic despite being 885-fold higher than the known topotecan IC_50_ for human RB cells (IC_50_ 14 ng/gm).^24^ Microneedle scleral patch platform offers the advantage of customizable dosing, enabling delivery of a defined drug quantity to achieve therapeutic (IC_50_) concentrations within the retina. The need for repeated applications or placement of multiple patches across different quadrants will be systematically evaluated in the next phase of our *in vivo* rabbit RB model.

A key limitation of this study is the lack of data on suprachoroidal injection or the amount of drug that could be injected in children with smaller eyes and thinner sclera (500 ± 36 μm).^66^^,^^67^ Our ongoing ultrasound biomicroscopy studies aim to characterize scleral thickness in treatment-naïve children with RB. The initial clinical application of MSP would require confirmation of sufficient scleral thickness and absence of choroidal tumor at the intended application site to ensure appropriate drug delivery. In addition, topotecan-loaded MSP may have limited drug distribution across quadrants due to the long posterior ciliary artery, which can be addressed by targeting the tumor quadrant or using 2 different application sites.^68^ Finally, tissue topotecan levels in nondisease-bearing rabbit eyes are not representative of tissue levels in the RB human eye. Although the data seem promising, ongoing efficacy studies in the rabbit RB model need to be demonstrated before initiating a phase I/II clinical study.

## Conclusions

Microneedle scleral patch fabricated using sodium hyaluronate has the physical attributes required for transscleral drug delivery in clinical applications. Microneedle scleral patch offers a promising alternative drug delivery route for RB treatment. A single topotecan-loaded MSP delivering 100 μg of topotecan achieved a retina-to-plasma concentration ratio of 275.7, indicating highly selective drug distribution to the retina. This ratio was 4.7-fold higher than that achieved by IAC (58.9) and over 209-fold higher than that achieved by IVC (1.32). Retinal topotecan concentrations reached levels up to 197-fold above the IC_50_ for human RB cells (14 ng/g) without evidence of toxicity. Increasing the dose to 100 μg topotecan resulted in similar nontoxic retinal concentrations, supporting a wide therapeutic window. These data demonstrate effective and selective retinal drug delivery via MSP, with potential clinical relevance for RB treatment.

## Patent Disclosure

A provisional patent application has been filed (Patent Application No. 202511074841, dated August 6, 2025) for the invention entitled “Microneedle Array, Applicator and Kit for Targeted Ocular Drug Delivery and Method of Preparation Thereof,” in the name of the Indian Council of Medical Research and L V Prasad Eye Institute.
