# Supplementary material for: Topotecan Microneedle Scleral Patch: A Transscleral Drug Delivery Study for Retinoblastoma
Source: Ophthalmol Sci. 2026 May 11;6(7):101226. doi: 10.1016/j.xops.2026.101226 (PMC13279026; doi:10.1016/j.xops.2026.101226)
Supplement: Figures S1 and S2 [file mmc1.pdf]

Supplementary materials

Topotecan microneedle scleral patch: A transscleral drug delivery study for retinoblastoma

Vishal Raval[1,2#], Arawindh R[1#], Sharayu Naik[1], Venkata Vamsi Krishna Venuganti[1]*

[1]Department of Pharmacy, Birla Institute of Technology and Science (BITS) Pilani, Hyderabad Campus, Hyderabad, India
[2]The Operation Eyesight Universal Institute for Eye Cancer, L V Prasad Eye Institute, Hyderabad, India

#Equal author contribution.

***Corresponding author:**

Venkata Vamsi Krishna Venuganti

Department of Pharmacy,

Birla Institute of Technology and Science (BITS) Pilani,

Hyderabad Campus, Hyderabad, India

Email: vamsi@hyderabad.bits-pilani.ac.in

Tel: +91-40-66303581

Abbreviated title: Topotecan microneedle scleral patch

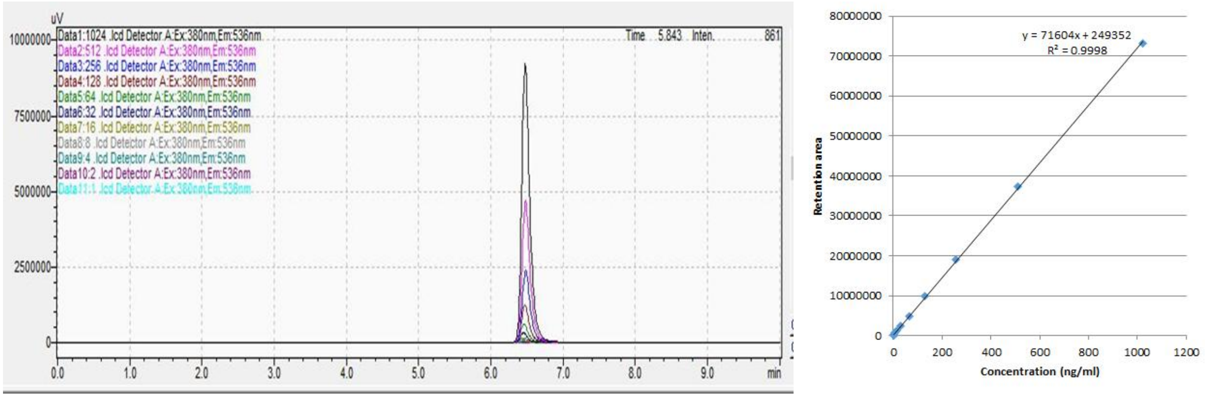

Figure S1. Representative overlay of the chromatogram of topotecan HCl and its calibration curve.

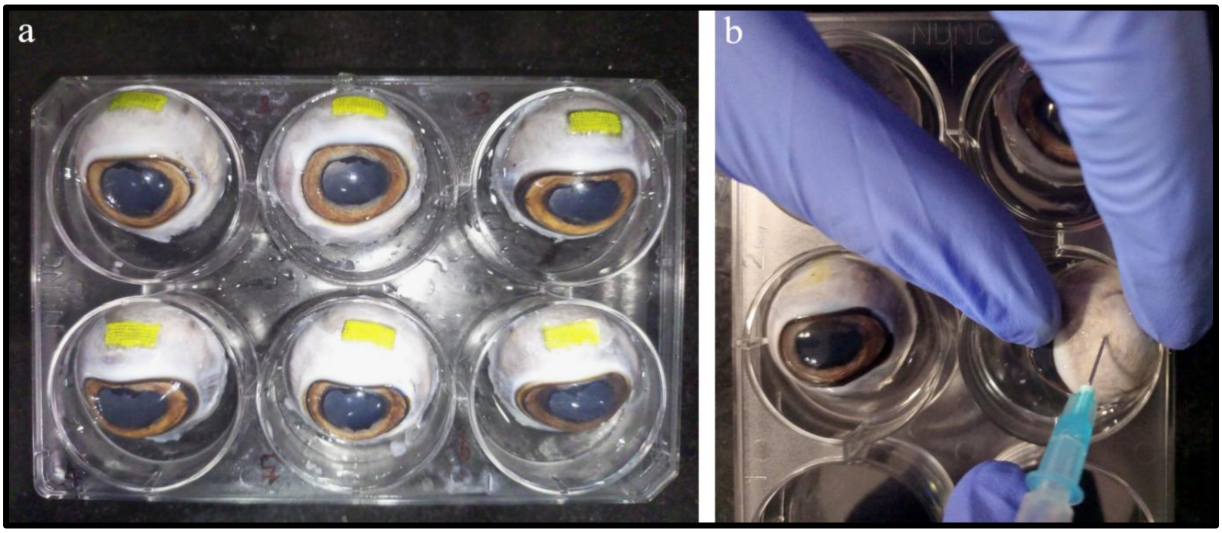

Figure S2. Experimental setup for *ex vivo* topotecan biodistribution study in excised goat eye globe after application of topotecan MSP (a) and intravitreal administration of topotecan (b).
